# Dexmedetomidine reduces enteric glial cell injury induced by intestinal ischaemia‐reperfusion injury through mitochondrial localization of TERT

**DOI:** 10.1111/jcmm.17261

**Published:** 2022-04-02

**Authors:** Qian Hu, Xiao‐Ming Liu, Zheng‐Ren Liu, Zhi‐Yi Liu, Huai‐Gen Zhang, Qin Zhang, Yuan‐Lu Huang, Qiu‐Hong Chen, Wen‐Xiang Wang, XueKang Zhang

**Affiliations:** ^1^ Department of Anesthesiology The First Affiliated Hospital of Nanchang University Nanchang China; ^2^ Department of Thoracic Surgery The First Affiliated Hospital of Nanchang University Nanchang China; ^3^ Department of General Surgery The First Affiliated Hospital of Nanchang University Nanchang China

**Keywords:** dexmedetomidine, enteric glial cells, intestinal ischaemia‐reperfusion injury, mitochondrial localization, oxidative stress, TERT

## Abstract

This study was performed to uncover the effects of dexmedetomidine on oxidative stress injury induced by mitochondrial localization of telomerase reverse transcriptase (TERT) in enteric glial cells (EGCs) following intestinal ischaemia‐reperfusion injury (IRI) in rat models. Following establishment of intestinal IRI models by superior mesenteric artery occlusion in Wistar rats, the expression and distribution patterns of TERT were detected. The IRI rats were subsequently treated with low or high doses of dexmedetomidine, followed by detection of ROS, MDA and GSH levels. Calcein cobalt and rhodamine 123 staining were also carried out to detect mitochondrial permeability transition pore (MPTP) and the mitochondrial membrane potential (MMP), respectively. Moreover, oxidative injury of mtDNA was determined, in addition to analyses of EGC viability and apoptosis. Intestinal tissues and mitochondria of EGCs were badly damaged in the intestinal IRI group. In addition, there was a reduction in mitochondrial localization of TERT, oxidative stress, whilst apoptosis of EGCs was increased and proliferation was decreased. On the other hand, administration of dexmedetomidine was associated with promotion of mitochondrial localization of TERT, whilst oxidative stress, MPTP and mtDNA in EGCs, and EGC apoptosis were all inhibited, and the MMP and EGC viability were both increased. A positive correlation was observed between different doses of dexmedetomidine and protective effects. Collectively, our findings highlighted the antioxidative effects of dexmedetomidine on EGCs following intestinal IRI, as dexmedetomidine alleviated mitochondrial damage by enhancing the mitochondrial localization of TERT.

## 1 INTRODUCTION

Intestinal ischaemia‐reperfusion injury (IRI) is a pathological phenomenon commonly occurring during vascular surgical procedures, such as abdominal aortic aneurysm repair and cardiopulmonary bypass.^1^ The sequelae of intestinal IRI trigger an increase in mitochondrial membrane permeability and bacterial translocation due to dysfunction of the intestinal epithelial barrier.^2^ Despite significant improvements in the arena of clinical therapy against IRI, its occurrence is still associated with high rates of mortality,^3^ with studies indicating excessively high mortality rates of 67%–80% in the critical care setting.^4^ Moreover, there is a lack of standardized therapeutic regimen with both high efficacy and safety to treat IRI.^5^ As one of the chief components of the enteric nervous system, enteric glial cells (EGCs) are well‐established crucial factor in the intestinal mucosal defence system.^6^ Furthermore, EGCs also play the pivotal role of protecting against the intestinal inflammatory response post‐IRI.^7^

The α2 adrenoceptor agonist, dexmedetomidine is well‐characterized as a sedative drug applied in clinical settings to treat patients suffering from anxiety and severe pain. Interestingly, dexmedetomidine was previously reported to exert a set of cardio‐protective effects in animal models following the induction of regional ischaemia.^8^ A prior double‐blind and dose‐escalation experiment highlighted that dexmedetomidine led to a decreased incidence of adverse cardiac events, including haemodynamic instability, in the ischaemic myocardium of patients who underwent open‐heart surgeries.^9^ Meanwhile, existing evidence highlights telomeres as DNA‐protein complexes capable of protecting chromosomes against genome instability events, whilst also being essential for the processes of cell division.^10^ During the course of DNA replication, the telomere sequences at the ends of the chromosomes are often lost, whereas those cells expressing telomerase are protected from the loss of telomeres.^11^ In the vast majority of normal human tissues, telomerase activity is generally undetectable; however, it is markedly upregulated in highly proliferative cells of neoplasms, bone marrow progenitor cells and the intestinal mucosa.^12^ What's noteworthy is that IRI was previously associated with increased telomerase activity in rat intestine samples, suggesting that dexmedetomidine treatment could repress IRI‐stimulated damage by downregulating the expression of telomerase and cleaved caspase‐3.^13^ On the other hand, the telomerase reverse transcriptase (TERT) gene encoding the catalytic subunit of TERT, represents a crucial component in the telomerase makeup.^14^ Accordingly, the current study sought to elucidate the effects associated with different doses of dexmedetomidine on intestinal IRI in rats. Furthermore, we aimed to investigate the mechanisms underlying the regulation of the expression of TERT and its mitochondrial localization as well as oxidative stress in EGCs of a rat model after administering dexmedetomidine, in an effort to uncover the potential treatment value of dexmedetomidine in regard to IRI.

## 2 MATERIALS AND METHODS

### 2.1 Ethics statement

The current study was performed with the approval of the Institutional Review Board of the First Affiliated Hospital of Nanchang University. Animal experiments were performed in accordance with the Guide for the Care and Use of Laboratory Animals published by the US National Institutes of Health. Extensive efforts were undertaken to minimize both the number and suffering of the experimental animals.

### 2.2 Establishment of intestinal IRI rat models

A total of 70 healthy adult male Wistar rats (weighing 280 ± 20 g) were procured and raised in a controlled netted cage environment under 25°C conditions. The rats were subjected to an initial acclimation period for one week prior to the commencement of the study with a 12 h day/night cycle and *ad libitum* access to water and food. Following anaesthesia induction, the rats were grouped into the sham group (*n* = 20, successful operation: death following operation failure =16:4), intestinal IRI group (*n* = 20, 10:10), low‐dose group (*n* = 20; 12:8; rats with intestinal IRI were injected with 10 μg/kg dexmedetomidine *via* tail vein) and high‐dose group (*n* = 20; 13:7; rats with intestinal IRI were injected with 100 μg/kg dexmedetomidine *via* tail vein).^15^, ^16^

The rats were anesthetized using an isoflurane vaporizer, after which the abdominal cavity was opened along the midline and the superior mesenteric artery (SMA) was exposed. Intestinal IRI rats were occluded by clamping the SMA with a miniature claw clip for 60 min. Two dexmedetomidine‐treated groups were administrated with dexmedetomidine at different concentrations 5 min prior to ischaemia, followed by a 60‐min period of reperfusion. The rats in the sham group were not subjected to SMA occlusion after the abdominal cavity was opened. After 1 h of blood flow recovery, the rats that were still breathing and exhibiting normal vital signs after all operations were regarded as successfully modelled. Subsequently, 10 IRI rats were randomly selected, anesthetized and then euthanized by means of carbon dioxide inhalation for 10 min. Following euthanasia, the small intestine (jejunum) of the rats was collected and placed on ice in order to remove the duodenum, followed by normal saline washing.^17^, ^18^

### 2.3 Haematoxylin and eosin (HE) staining

The obtained small intestinal tissues were fixed with 10% neutral formaldehyde solution for more than 24 h, dehydrated with an ascending series of alcohol (70%, 80%, 90%, 95%, 100%, 1 min/time), cleared twice in xylene, 5 min per time and paraffin‐embedded. Subsequently, the tissues were sliced into 5 μm‐thick section and stained with haematoxylin (H8070‐5 g, Beijing Solarbio Life Sciences Co., Ltd.,) for 4 min and eosin (PT001, Shanghai Bogoo Biological Technology Co., Ltd.,) for 2 min.^19^ Staining results were visualized under a light microscope (DMM‐300D, Shanghai Caikon Optical Instrument Co., Ltd.,). Intestinal tissue sections were scored using a semiquantitative grading system to determine the extent of mucosal damage.^20^

### 2.4 Immunohistochemistry (IHC)

About 3 cm of rat's small intestine (jejunum) was selected, rinsed with pre‐cooled normal saline and dried with paper towels. Next, the intestinal tissues were fixed with 10% formalin for two days, paraffin‐embedded and sliced into 4 μm‐thick sections. The obtained sections were sequentially rinsed with 3% H_2_O_2_ for 10 min and reacted with a blocking solution comprised of 10% goat serum (Cwbiotech Co., Ltd.,) at room temperature for 20 min. Primary antibody against TERT (1: 50, PA5‐116024, Invitrogen Inc.,) and secondary antibody biotin‐labelled goat anti‐rabbit immunoglobulin G (IgG; 1: 1000, ab6721, Abcam, Cambridge, UK) were adopted. IHC analysis of TERT was performed based on a previously published method.^21^

### 2.5 RNA isolation and quantitation

Total RNA content was extracted from the tissues using RNA extraction kits (D203‐01, GenStar Biosolutions,). Reverse transcription procedures were performed with the help of PrimeScript RT reagent kits (RR047A, Takara Holdings Inc.,). The primers were designed and synthesized by Takara Holdings Inc. (listed in Table S1). Reverse transcription‐quantitative polymerase chain reaction (RT‐qPCR) was carried out with RT‐qPCR kits (Q511‐02, Vazyme Biotech,) on a Bio‐Rad's CFX96 real‐time system. The relative expression of each target gene was subsequently calculated based on the 2^−ΔΔCt^ method, with glyceraldehyde‐3‐phosphate dehydrogenase (GAPDH) serving as the loading control.

### 2.6 Western blot analysis

Total cell lysates were prepared on ice. The Western blot analysis procedure was performed based on a previously published method.^22^ For immunoblotting of targeted proteins, the following primary antibodies were used: p65 (rabbit monoclonal antibody, 1: 5000, ab32536, Abcam), TERT (rabbit polyclonal antibody, 1: 1000, PA5‐116024, Invitrogen), p53 (mouse monoclonal antibody, 1: 200, ab90363, Abcam), Bcl‐2 (mouse monoclonal antibody, 1: 500, ab692, Abcam), MnSOD (rabbit polyclonal antibody, 1: 5000, ab13533, Abcam), Bax (rabbit polyclonal antibody, 1: 1000, ab32503, Abcam), AIF (rabbit polyclonal antibody, 0.25 μg/mL, ab1998, Abcam), S100β (rabbit monoclonal antibody, 1: 1000, ab52642, Abcam), GFAP (rabbit monoclonal antibody, 1: 2000, ab33922, Abcam), caspase‐3 (rabbit polyclonal antibody, 1: 500, ab13847, Abcam) and GAPDH (ab9485, Abcam). Additionally, the horseradish peroxidase (HRP)‐conjugated secondary antibody goat anti‐rabbit IgG (1: 5000, ab6721, Abcam) or goat anti‐mouse IgG (1: 5000, ab6728, Abcam) were also adopted. The relative expression of proteins was normalized to that of GAPDH.

### 2.7 Isolation of rat EGCs

The isolation of rat EGCs was carried out with reference to a previously published literature.^23^ Briefly, about 7 cm of the small intestine (jejunum) segment of the E17‐19 foetal mice was removed from the gastrointestinal segment, and pre‐chilled DPBS was adopted to flush the intestine (jejunum) with a 5 mL syringe. Subsequently, the small intestine (jejunum) was sliced into approximately 3 cm, and the muscle tissues and mesentery attached to the outer wall of the intestine (jejunum) were carefully removed with the tip of a syringe. Thereafter, a clean pair of scissors were used to slice the small intestine (jejunum) into sections of about 0.5 cm and then placed in 30 mL pre‐cooled EDTA/HEPES/DPBS buffer, followed by incubation by shaking at 4°C for 10 min. Next, the intestinal (jejunum) sections were dispersed with a pipette to isolate the cells from the epithelial layer of the gastric mucosa. Following filtration through a 100 μm nylon membrane cell filter, the filter membrane was eluted with the cell recovery solution at 4°C for 25–30 min. After elution, the cells were placed in DMEM containing 10% foetal bovine serum, 500 μm/L glutamine, penicillin and streptomycin and cultured at a density of 4 × 10^4^ cells/cm^2^. At the second week of culture, the cultured cells were identified by means of GFAP immunofluorescence staining.

### 2.8 Transmission electron microscopy (TEM) and Flameng grading

The mitochondrial morphology of cells was visualized using an H‐7650 TEM (Hitachi,), with the degree and extent of mitochondrial damage evaluated based on the Flameng scores.^24^, ^25^ A total of 5 visual fields were randomly selected from each sample, with the mitochondrial structural damage in each visual field evaluated accordingly. The average scores for the mitochondrial morphology and structure in each sample were calculated.

### 2.9 Immunofluorescence staining

The cells were inoculated into a 6‐well plate in an independent fashion. Upon achieving 50% confluence, the cells were subsequently cultured with complete medium and treated with 400 μM H_2_O_2_ for 3 h or 100 μM pyrrolidine dithiocarbamate (PDTC) for 24 h, followed by treatment with 400 μM H_2_O_2_ for 3 h.

Afterwards, the cells were rinsed with PBS and incubated with HBSS (Gibco) containing 200 nM MitoTrackerRed (C1049B, Beyotime Biotechnology Co.,) at 37°C for 30 min. Following three rinses with PBS, the cells were fixed with 4% pre‐cooled paraformaldehyde for 15 min, treated with 0.2% Triton X‐100 for 5 min, blocked with 3% goat serum for 30 min and probed with the diluted primary antibody TERT (PA5‐116024, 1: 100, Invitrogen) at 4°C overnight. The following day, the cells were re‐probed with secondary antibody (ab150077, 1: 300, Abcam) for 1 h at room temperature, stained with DAPI (C1006, Beyotime), mounted and scanned with a laser confocal microscope (FV1000, Olympus Optical Co., Ltd.,), with the fluorescence images documented.

### 2.10 Isolation of cell nucleus and mitochondrion

Initially, 1 × 10^7^ cells were collected and added with 2 mL of pre‐cooled 0.25 mol/L sucrose and 0.003 mol/L calcium chloride solution, after which the mixture was subjected to 10 rounds of grinding and subsequently centrifuged for 15 min at 2500 rpm. Next, the supernatant was transferred to a high‐speed centrifugal tube and stored in a beaker with ice cubes for mitochondria isolation. The remaining precipitates were suspended in 2 mL of 0.25 mol/L sucrose and 0.003 mol/L calcium chloride solution and centrifuged at 2500 rpm for 15 min. The supernatant was subsequently discarded, after which the precipitates were added with protein lysis buffer to obtain the nuclear protein. The high‐speed centrifugal tube containing the supernatant was then centrifuged at 17,000 rpm for 20 min after balancing, followed by removal of the supernatant. The precipitates were then added with 1 mL of 0.25 mol/L sucrose and 0.003 mol/L calcium chloride solution. Afterwards, suspension preparation was conducted using a pipette and centrifuged at 17,000 rpm for 20 min, after which the supernatant was discarded, added with the protein lysis buffer and followed by mitochondrial protein collection.

### 2.11 Measurement of reactive oxygen species (ROS), glutathione (GSH) and malondialdehyde (MDA)

ROS levels in cells were determined with the help of reactive oxygen detection kits (S0033S, Beyotime), after which the results were analysed using a flow cytometer (CytoFLEX, Beckman Coulter Inc.,)^26^ at an excitation wavelength of 488 nm and an emission wavelength of 525 nm. Meanwhile, GSH and MDA contents were determined by means of DTNB Kit (S0055, Beyotime) and MDA Kit (S0131S, Beyotime), respectively.^27^, ^28^

### 2.12 Determination of mitochondrial permeability transition pore (MPTP)

MPTP was determined in accordance with the instructions of the MPTP kit (C2009S, Beyotime). Briefly, the cells were rinsed with PBS, added with an appropriate volume of calcein AM staining solution, fluorescence quenching working solution or Ionomycin control, shaken gently to make the dye evenly cover all cells and incubated at 37°C for 30 min in conditions void of light. Subsequently, the cells were cultured in fresh 37°C preheated complete medium and then incubated at 37°C for 30 min in dark conditions to ensure that the intracellular esterase fully hydrolysed calcein AM to generate calcein with green fluorescence. Next, the cells were rinsed with PBS and observed under a fluorescence microscope (BX63, Olympus) at an excitation wavelength of 494 nm and an emission wavelength of 515 nm.

### 2.13 Determination of mitochondrial membrane potential (MMP)

The MMP was determined with the help of MMP kits (C2008S, Beyotime), in accordance with the manufacturer's instructions. In short, the cells were rinsed with PBS, added with 1 mL rhodamine 123 staining working solution, incubated at 37°C for 30 min in a cell incubator. Subsequently, the cells were rinsed twice with fresh pre‐warmed complete medium at 37°C and observed under a fluorescence microscope (BX63, Olympus) at an excitation wavelength of 507 nm and an emission wavelength of 529 nm.

### 2.14 Detection of oxidative mtDNA damage and common mtDNA deletions

8‐hydroxy‐2′‐deoxyguanosine (8‐OHdG) can be adopted as a marker of the mitochondrial damage process. RT‐qPCR was performed to detect the content of 8‐OHdG in mitochondrial DNA. Mitochondrial DNA Isolation kits (LS‐K839, Lifespan Biosciences) were utilized to extract mtDNA from cells. The extracted mtDNA was divided into two portions, 1 μL of which was incubated with 1 U hOGG1 enzyme in 1 × NE buffer (20 mM Tris chloride, pH 8.0; 1 mM EDTA; 1 mM DDT and 100 μg/mL BSA) at 37°C for 1 h, and then at 65°C for 20 min. The remaining portion was treated with hOGG1‐free enzyme. Since hOGG1 enzyme can cut DNA fragments containing 8‐OHdG, which further reduces the PCR amplification efficiency of the fragments after hOGG1 enzyme treatment, RT‐qPCR was carried out to compare CT1 obtained by amplifying mtDNA fragments after hOGG1 enzyme treatment, and the CT2 obtained by amplifying mtDNA fragment without hOGG1 enzyme treatment. Afterwards, the degree of mtDNA damage was expressed as the ratio of CT1/CT2. Larger CT1/CT2 value reflected the smaller mitochondrial damage, and vice versa.

The primer sequences used in this experiment to amplify mtDNA fragments were as follows: mtF3212, 5'‐CACCCAAGAACAGGGTTTGT‐3’, mtR3319, 5'‐TGGCCATGGGATTGTTGTTAA‐3'. The relevant sequences for detection of mitochondrial common mutation levels are illustrated in Table S2.

Mitochondria tend to produce various mutations upon damage, and some of these mutation types are often common in numerous types of mitochondrial damaged cells. Amongst them, 4977bp deletion mutation as a common mtDNA deletion mutation can be adopted to detect the degree of mitochondrial damage. Meanwhile, the level of mitochondrial common deletion mutations (mtDNA 4977bp deletion) can be determined using the ratio of the 4977bp deletion of mitochondrial DNA content to the total mitochondrial DNA content by RT‐qPCR. The content of mitochondrial DNA with 4977bp deletion can be detected with the mtDNA_4977bp primer to determine the CT value of the sample and refer to the standard curve to obtain the specific value. More so, the total mitochondrial DNA content can be detected by the COX I primer to determine the sample CT value and refer to the standard curve to obtain the specific value. Total nuclear DNA content was detected with a β‐actin primer to determine the CT value of the sample and refer to the standard curve to obtain the specific value. At the same time, the level of mitochondrial common deletion mutations was calculated using the following formula: mitochondrial content =total mitochondrial DNA/total nuclear DNA; 4977bp deletion mitochondrial DNA/mitochondrial content.

### 2.15 Cell Counting Kit‐8 (CCK‐8) assay

Cells were inoculated into a 96‐well plate, at a density of 6 × 10^3^ cells/well, to obtain a final volume of 200 μL of cells/well, and then maintained in 100 μL of a mixture of CCK‐8 solution (CA1210, Solarbio) and serum‐free medium at a ratio of 1: 9 at 37°C for 2 h in conditions void of light. The absorbance values were detected at 490 nm using an enzyme labelling instrument. Six replicate wells were established to obtain the mean value.

### 2.16 5‐ethynyl‐2’‐deoxyuridine (EdU)

Cell proliferation of cells was detected with the help of EdU Apollo DNA in vitro kits (C10310‐2, Guangzhou RiboBio Co., Ltd.,), in accordance with the manufacturer's instructions. Primarily, each well of cells was incubated with EdU medium for 2 h, then cultured with PBS containing 4% paraformaldehyde for 30 min, glycine for 10 min and then permeabilized with the permeabilizing reagent. Next, the cells were added with 1 × Apollo^®^ staining reaction solution and incubated for 30 min in conditions void of light and then added with 1 × Hoechst reaction solution and incubated for 10 min in dark conditions. Lastly, the cells were rinsed with PBS, and cell proliferation was detected by means of flow cytometry.

### 2.17 Flow cytometry

Cell apoptosis was detected with the help of Annexin V‐FITC/propidium iodide (PI) double staining kits (CA1020, Solarbio). After 48 h of culture, the cells were collected and detached with 0.25% trypsin solution. Following centrifugation, the cells were resuspended in 200 μL of binding buffer, followed by the addition of 10 μL of Annexin V‐FITC and 5 μL of PI. Subsequently, the cells were added 300 μL of binding buffer. Afterwards, red fluorescence was observed at 488 nm and recorded using a flow cytometer (6HT, Cellwar Biotechnology Co., Ltd.,) to determine cell apoptosis.

### 2.18 Statistical analysis

All experimental data were processed and analysed using the SPSS 22.0 statistical software (IBM Corp.). Measurement data were presented as mean ± standard deviations. Comparisons of data amongst multiple groups were carried out using a one‐way analysis of variance (ANOVA), and comparisons between two groups were analysed using the least significant difference (LSD) test. Values recorded at different time points were compared using repeated‐measures ANOVA. A *p* value <0.05 was regarded statistically significant.

## 3 RESULTS

### 3.1 Dexmedetomidine treatment protected rats against intestinal IRI

**FIGURE 1:**
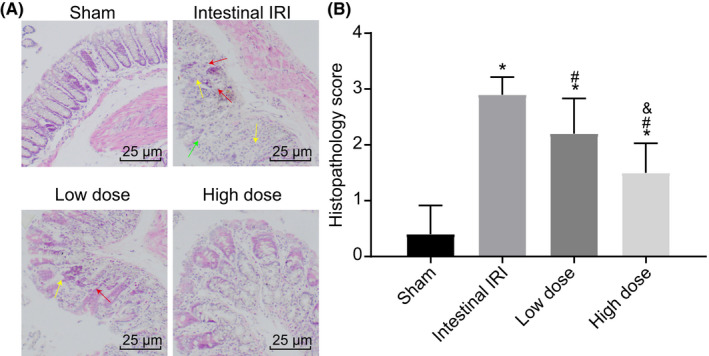
Dexmedetomidine reduces intestinal IRI in rats. (A) Images of HE staining depicting the pathological changes in small intestinal tissues of rats from each group (× 400, scale bar =25 μm). (B) Histopathology scores for the injury of the small intestinal tissue in each group. *, *p* < 0.05 compared with the sham group; #, *p* < 0.05 compared with the intestinal IRI group; and &, *p* < 0.05 compared with the low‐dose group. The measurement data were statistically analysed and presented as the mean ± standard deviation; data were compared using one‐way ANOVA. *n* = 10. EGCs, enteric glial cells; IRI, ischaemia‐reperfusion injury; HE, haematoxylin and eosin; n, number

Intestinal IRI is a common life‐threatening complication that can present with the advent of mesenteric artery thrombosis, strangulated intestinal obstruction, trauma, abdominal aortic aneurysm surgery and bowel transplantation.^29^ Therefore, it is imperative to improve the current means of alleviating intestinal IRI. Existing evidence suggests that dexmedetomidine confers a protective effect on IRI in coronary artery endothelial dysfunction in heart.^30^ To elucidate whether dexmedetomidine also exerts a protective effect against intestinal IRI, intestinal IRI rat models were established. In accordance with the observations made under a light microscope, the results of HE staining illustrated that the intestinal mucosa in the sham group did not exhibit any notable pathological changes; the intestinal villi were clearly visible with a small number of infiltrated inflammatory cells with very few necrotic epithelial cells detected (Figure 1A). Meanwhile, in the intestinal IRI group, the intestinal mucosa exhibited varying degrees of damage. Shortened villi (green arrows), intestinal mucosal haemorrhaging and oedema, inflammatory cell infiltration (red arrows), and damaged and necrotic epithelial cells with a mild degree of intestinal mucosal gland dilation (yellow arrows) were all documented (Figure 1B). In comparison to the intestinal IRI group, the low‐dose and high‐dose groups exhibited lesser degree of intestinal mucosal injury and inflammatory exudation, accompanied by a smaller number of damaged cells, and a lesser degree of significant damage. The increase in drug dosage was positively correlated with a reduction in damage, as indicated by the decrease in structural disturbances. Together, these findings preliminarily suggested that dexmedetomidine treatment could alleviate intestinal IRI.

### 3.2 Dexmedetomidine pretreatment increased EGC viability and inhibited EGC apoptosis

**FIGURE 2:**
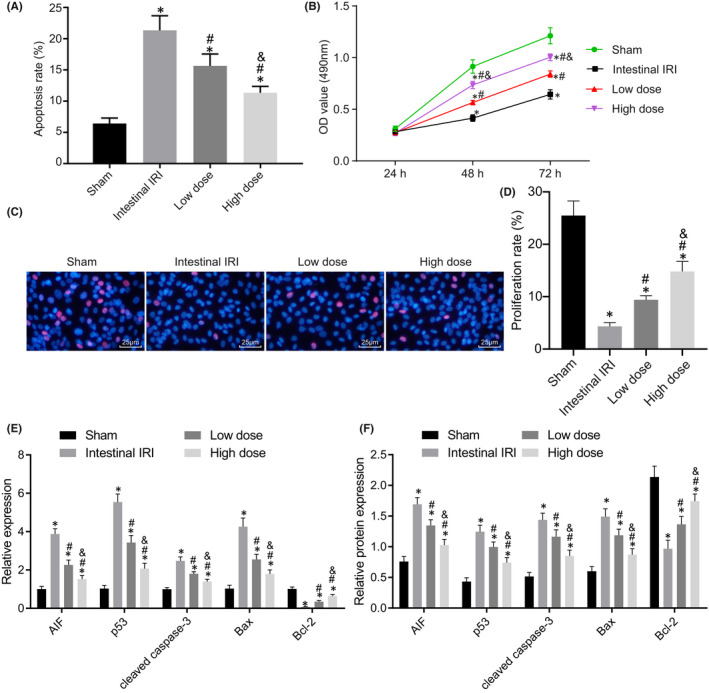
Dexmedetomidine increases EGC viability and inhibits EGC apoptosis. (A) EGC apoptosis is determined by Annexin V‐FITC/PI. (B) EGC proliferation determined by CCK‐8. (C) EGC proliferation is determined by EdU (× 400, scale bar =25 μm). (D) Statistics of panel C. E, RT‐qPCR detection of p53, cleaved caspase‐3, AIF, Bax and Bcl‐2 expression in EGCs. F, Western blot analysis of p53, cleaved caspase‐3, AIF, Bax and Bcl‐2 proteins in EGCs. *, *p* < 0.05 compared with the sham group; #, *p* < 0.05 compared with the intestinal IRI group; and &, *p* < 0.05 compared with the low‐dose group. The measurement data were statistically analysed and presented as the mean ± standard deviation; data were compared using one‐way ANOVA. The cell experiment was repeated 3 times

Intestinal IRI often damages the intestinal epithelial barrier, rendering it dysfunctional. As an important part of the intestinal epithelial barrier, EGCs play a major role in maintaining the integrity of the intestinal epithelial barrier.^6^ Accordingly, we examined the effects of dexmedetomidine on the proliferation and apoptosis of EGCs. The results of Annexin V‐FITC/PI double staining data demonstrated that EGC apoptosis was enhanced in the intestinal IRI group compared to that in the sham group, whilst being decreased in the dexmedetomidine group in a dose‐dependent manner (Figure 2A). Moreover, CCK‐8 and EdU assays results further illustrated that the proliferation of EGCs was significantly decreased in the intestinal IRI group compared to that in the sham group. In addition, the proliferation of EGCs was increased dramatically after dexmedetomidine pretreatment, in a dose‐dependent manner (Figure 2B–D).

Furthermore, the results of RT‐qPCR and Western blot analysis revealed that compared with the sham group, the remaining groups exhibited significantly increased expression levels of p53, cleaved caspase‐3, AIF and Bax, yet those of Bcl‐2 were decreased in EGCs. Compared with the intestinal IRI group, the low‐dose group presented with reduced expression levels of p53, cleaved caspase‐3, AIF and Bax and increased expressions of Bcl‐2. Relative to the low‐dose group, the high‐dose group exhibited a more pronounced decline in the expressions of p53, cleaved caspase‐3, AIF and Bax, and a more obvious enhancement in the expression of Bcl‐2 (Figure 2E,F, Figure S1A). Overall, these findings indicated that dexmedetomidine could reduce EGC apoptosis caused by intestinal IRI.

### 3.3 Dexmedetomidine treatment upregulated TERT expression and caused accumulation of TERT protein within EGC mitochondria

Existing evidence indicates that TERT deficiency can delay functional recovery after IRI,^31^, ^32^ whilst exogenous human telomerase reverse transcriptase gene exerts a protective effect on IRI in aged liver donor.^33^ Moreover, intestinal IRI was previously shown to be primarily caused by ROS,^17^ whilst antioxidant treatment can attenuate apoptosis during IRI.^34^ Owing to the fact that the mitochondrial respiratory chain produces large amounts of ROS, we speculated whether TERT might be related to mitochondria function during IRI.

**FIGURE 3:**
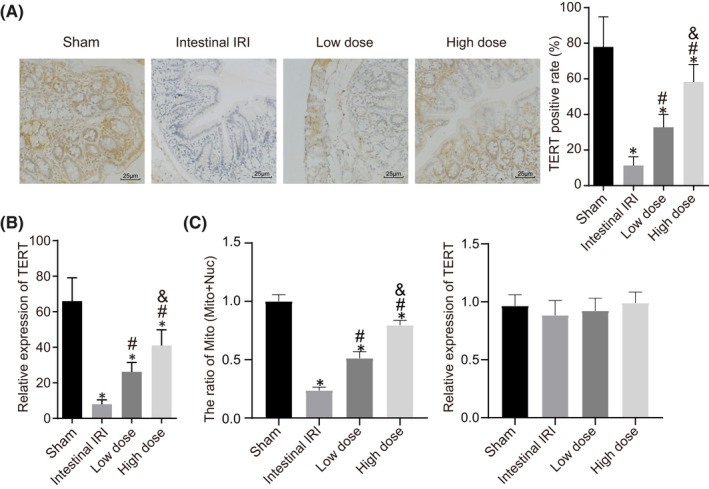
Dexmedetomidine promotes TERT levels and accumulation of TERT protein within EGC mitochondria. (A) IHC staining of TERT protein in small intestinal tissues of rats (× 200). (B) Immunofluorescence staining of TERT expression and localization in EGCs in rats from each group. (C) Western blot analysis of TERT protein in the isolated mitochondria from primary EGCs wherein VDAC1 was an internal reference. *, *p* < 0.05 compared with the sham group; #, *p* < 0.05 compared with the intestinal IRI group; and &, *p* < 0.05 compared with the low‐dose group. The measurement data were statistically analysed and presented as the mean ± standard deviation; data were compared using one‐way ANOVA. The cell experiment was repeated 3 times. *n* = 10

Subsequent results of IHC staining illustrated that TERT was predominantly located in the nucleus and was strongly positively expressed in the intestinal tissues of rats in the sham group. Relative to the sham group, the intestinal IRI group presented with less than 5% of TERT‐positive cells and TERT was expressed in the nucleus. Compared with the intestinal IRI group, the dexmedetomidine groups exhibited increased TERT‐positive cells, being more localized in the cytoplasm showing a dose‐dependent manner (Figure 3A).

Furthermore, the expression and distribution patterns of TERT in EGCs were observed by means of immunofluorescence staining. Compared with the sham group, TERT fluorescence was reduced in the EGCs of the intestinal IRI group, showing no difference in co‐localization with mitochondria. However, the pretreatment with dexmedetomidine augmented both the TERT fluorescence in EGCs and the co‐localization with mitochondria in a dose‐dependent manner (Figure 3B, Figure S2).

Subsequently, mitochondria and nuclear proteins were isolated to further detect the expression and localization patterns of TERT. The results illustrated that TERT protein expression level in the EGC mitochondria was decreased in the intestinal IRI group relative to those in the sham group. However, opposite trends were observed in TERT protein expression levels following dexmedetomidine pretreatment, showing a dose‐dependent manner, whereas there were no significant changes in the total TERT protein expression in the cells (Figure 3C, Figure S1B). Together, these findings indicated that dexmedetomidine pretreatment could promote both TERT expression and its mitochondrial localization in EGCs.

### 3.4 Dexmedetomidine treatment abolished the effect of intestinal IRI on the mitochondrial morphology and function of EGCs

**FIGURE 4:**
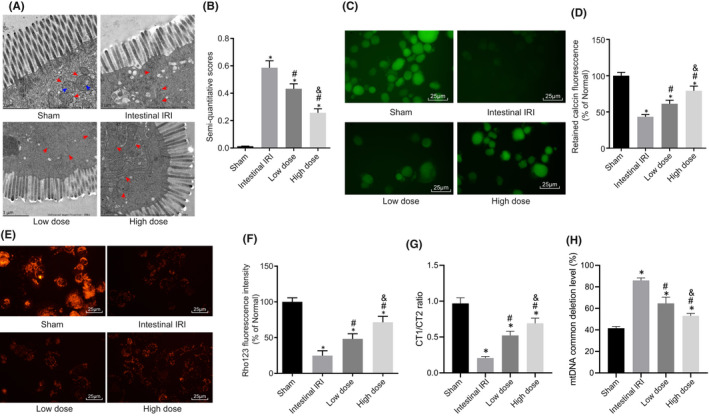
Dexmedetomidine reduces changes in the mitochondrial morphology and functions in EGCs. (A) TEM images of the mitochondria in primary EGCs in each group. (B) Semi‐quantitative scores of EGCs. (C) Mitochondrial membrane staining of EGCs in each group (scale bar =25 μm). (D) Level of MPTP in each group of EGCs. (E) Mitochondrial membrane staining of EGCs in each group (scale bar =25 μm). (F) Level of MMP in each group of EGCs. (G) Level of mitochondrial mtDNA damage in each group of EGCs. (H) Statistics of mitochondrial common deletion mutation levels in each group. *, *p* < 0.05 compared with the normal, oe‐NC or sh‐NC group; #, *p* < 0.05 compared with the intestinal IRI group; and &, *p* < 0.05 compared with the low‐dose group. The measurement data were presented as the mean ± standard deviation; data were compared using one‐way ANOVA. The cell experiment was repeated 3 times. *n* = 10

TEM was employed to observe the changes in mitochondrial morphology in EGCs, which illustrated that mitochondrial morphology in EGCs in the sham group was normal (red arrows), with clear and intact cell membrane, homogeneous matrix, dense mitochondrial cristae, clear cell boundaries and smooth myofilaments (blue arrows). Relative to the sham group, the intestinal IRI group exhibited some swollen mitochondria, vacuolar changes, absence of mitochondrial cristae, blurred inner and outer membranes, destroyed EGC mitochondria fibres, decreased matrix density, partial matrix coagulation and ruptured mitochondrial cristae. Meanwhile, in the low‐dose group, there were a few swollen mitochondria, blurred mitochondrial cristae, intact inner and outer membranes, and no significant damage to the mitochondria. However, there was an improvement in regard to mitochondrial damage in the high‐dose group (Figure 4A). Compared with the sham group, significantly higher semiquantitative scores for EGCs were recorded in the intestinal IRI group, and lower scores were recorded in the low‐dose and high‐dose groups, of which the high‐dose group exhibited more pronounced declined scores (Figure 4B).

Compared with the sham group, the mitochondrial membrane staining of the EGCs of the other groups was much lighter, indicating destruction of mitochondrial membrane, and a significant increase in MPTP. Relative to the intestinal IRI group, the mitochondrial membrane staining of the EGCs of the dexmedetomidine groups was darkened and MPTP was decreased (Figure 4C,D). Meanwhile, MMP in EGCs was decreased in the intestinal IRI group compared to that in the sham group, suggestive of mitochondrial membrane destruction. Overall, these findings indicated that dexmedetomidine treatment could increase MMP in a dose‐dependent manner (Figure 4E,F).

Furthermore, the results of RT‐qPCR illustrated lower ratio of CT1/CT2 in EGCs in the intestinal IRI group compared to the sham group. Conversely, the ratio of CT1/CT2 was increased in the dexmedetomidine groups relative to the intestinal IRI group, with the high‐dose group showing a much higher ratio (Figure 4G). These findings indicated that dexmedetomidine could effectively reduce the level of mitochondrial damage in EGCs.

Furthermore, the common mutation level of 4977bp deletion in the mitochondria of EGCs was increased in the intestinal IRI group relative to that in the sham group. In the dexmedetomidine groups, 4977bp deletion levels were reduced and the high‐dose group showed a more profound reduction (Figure 4H). Altogether, the above‐mentioned findings indicated that dexmedetomidine could significantly inhibit common 4977bp deletion mutations in mitochondria, and as the dose increased, dexmedetomidine further inhibits mitochondrial deletion mutations and protects mitochondrial DNA.

### 3.5 Dexmedetomidine impaired oxidative stress in EGCs

**FIGURE 5:**
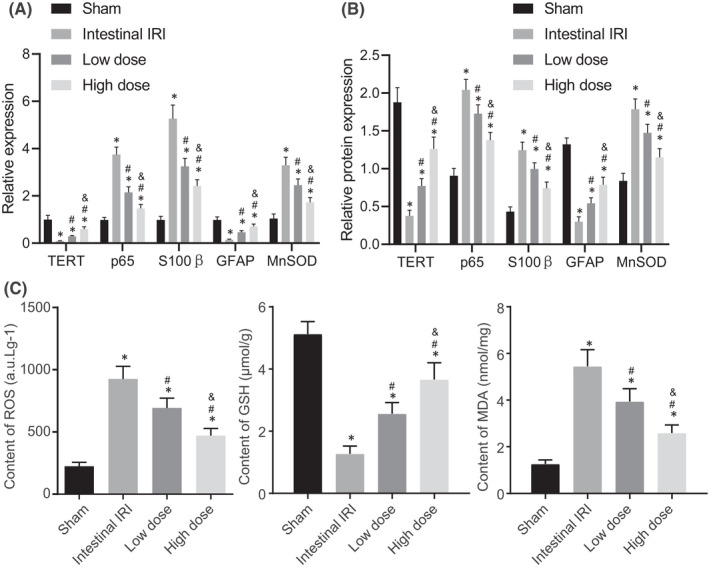
Dexmedetomidine suppresses oxidative stress following intestinal IRI. (A) The mRNA expressions of TERT, p65, MnSOD, S100β and GFAP in EGCs were determined by RT‐qPCR. (B) The protein expressions of TERT, p65, MnSOD, S100β and GFAP in EGCs were assessed by Western blot analyses. (C) Determination of the levels of ROS, GSH and MDA in EGCs. *, *p* < 0.05 compared with the sham group; #, *p* < 0.05 compared with the intestinal IRI group; and &, *p* < 0.05 compared with the low‐dose group. The measurement data were presented as the mean ± standard deviation and compared using one‐way ANOVA. The cell experiment was repeated 3 times

As indicated by the above‐mentioned results, dexmedetomidine could protect the mitochondrial morphology and function of EGC cells from IRI. Accordingly, we explored how dexmedetomidine protects the function of mitochondria. Since mitochondria play an important role in oxidative stress, the expression patterns of oxidative stress‐related genes (p65, S100β and MnSOD) and the neurogliocyte marker GFAP, and the levels of oxidative stress‐related factors (ROS, GSH and MDA), all of which can reflect the oxidative‐reduction state and oxidative damage in cells were measured. The collective results of RT‐qPCR and Western blot analysis revealed that, compared with the sham group, the other groups exhibited significantly increased expression levels of TERT, p65, MnSOD and S100β, in addition to reduced expressions of GFAP in EGCs; meanwhile, the low‐dose group presented with decreased expression levels of TERT, p65, MnSOD and S100β, along with increased expressions of GFAP in EGCs compared with those in the intestinal IRI group, whilst these results were more pronounced in the high‐dose group (Figure 5A,B, Figure S1C).

As shown in Figure 5C, the intestinal IRI group exhibited higher levels of ROS and MDA and lower GSH levels in EGCs relative to those in the sham group. Compared with the intestinal IRI group, the dexmedetomidine groups presented with lower ROS and MDA levels and higher GSH levels in EGCs. Moreover, ROS and MDA levels were further decreased whilst GSH levels were substantiated in the high‐dose group. Simultaneously, the above experiments were repeated in the intestinal tissues of rats with IRI and the experimental results were consistent with the cell experiment results (Figure S3A–C). Overall, these findings indicated that dexmedetomidine significantly inhibited oxidative stress following intestinal IRI.

## 4 DISCUSSION

The prime focus of the current study was to ascertain as to whether dexmedetomidine could protect EGCs from intestinal IRI. Subsequent experimentation revealed that an appropriate dosage of dexmedetomidine could mediate the mitochondrial localization of TERT and oxidative stress. More so, dexmedetomidine conferred an alleviating effect on intestinal IRI and protected EGCs *via* inhibition of mitochondrial localization of TERT and oxidative stress.

Intestinal IRI is often associated with intestinal epithelial barrier (IEB) dysfunction, whereas the hard‐done work of our peers suggests that EGCs play an important role in the maintenance of functional integrity of IEB.^35^, ^36^ Meanwhile, prior studies have also documented the involvement of GDNF in the barrier induction of intestinal epithelial cells by EGCs stimulated by acute ischaemia‐reperfusion^6^ It is also noteworthy that ischaemia and reperfusion confer various effects on EGCs and contractile activity of ileum.^37^ Herein, findings in our study illustrated the presence of decreased TERT and increased mitochondrial localization in the EGCs of rats with intestinal IRI. The telomerase ribonucleoprotein comprises of several subunits, including the TERT, telomerase RNA component (TERC), dyskerin pseudouridine synthase 1 (DKC1) and TEP1 subunits. Existing literature has indicated that the TERT subunit is homologous to a protein known as Est2p from *Saccharomyces cerevisiae*, which serves as a necessary factor for telomere maintenance.^38^ Research over the past two decades has further underscored the functions of TERT in addition to its main function in telomere maintenance.^39^ Particularly, TERT downregulation was previously shown to diminish embryonic stem cell proliferation, increase the number of cells at the G1 phase and decrease the number of cells at the S phase. More importantly, downregulation of TERT brought about a loss of cell pluripotency and human ESC differentiation to extraembryonic and embryonic lineages.^39^, ^40^ Furthermore, another study unveiled that TERT could protect human lung fibroblasts from apoptosis induced by oxidative stress and cultured neural cells from DNA‐damaging agent‐induced cell death.^41^ Oxidative stress can lead to TERT translocation from the nucleus to the mitochondria, where it shields the mitochondria, ultimately conferring resistance to apoptosis and exerting its telomere‐independent functions.^42^ Similarly, Li et al. previously uncovered that TERT was capable of protecting mtDNA, thereby serving as an indirect indicator of mitochondrial function, from damaged caused by oxidative stress in vitro and in vivo.^43^ Our findings provided evidence such that, mtDNA levels were decreased by dexmedetomidine‐mediated increase in TERT levels.

Additionally, recent discoveries have highlighted the emphasis on lower mtDNA levels and systemic mitochondrial defects in TERT knockout mouse models.^44^ Similarly, a prior study demonstrated that TERT binding to mtDNA could protect cells from ethidium bromide‐induced mitochondrial dysfunction.^45^ What's more, the increased MPTP induced by intestinal IRI could be diminished by dexmedetomidine in a dose‐dependent manner, whereas the opposite was true for downregulation of MMP induced by intestinal IRI in regard to dose‐dependent dexmedetomidine. MPTP and MMP are both well‐established as crucial indicators of mitochondrial function.^46^, ^47^ Further, elevated MPTP and reduced MMP were previously indicated as a sign of reperfusion injury.^48^ Overall, it would be plausible to suggest that dexmedetomidine treatment could reverse the adverse effects of intestinal IRI on mitochondrial function.

Another key discovery of our study was that dexmedetomidine could diminish mitochondrial localization and oxidative stress. Interestingly, dexmedetomidine was previously highlighted to attenuate renal cell injury induced by kidney IRI and also confer protection on the kidney from renal injury.^49^ In addition to the kidney, dexmedetomidine is also known to exert an antioxidant effect on the brain and heart.^50^ What's more, our findings were further corroborated by the study conducted by Ammar et al. showing that dexmedetomidine diminished the extent of cardiac and renal injury, as evidenced by decreased levels of myocardial‐specific proteins, kidney‐specific proteins and inflammatory cytokines.^51^ A prior study further illustrated that dexmedetomidine exerted its neuroprotective effects by either mediating the balance between proapoptotic and antiapoptotic factors, or by preconditioning against ischaemic injury.^52^ Meanwhile, IRI is known to precipitate increased levels of malondialdehyde, which is an end product of lipid peroxidation, and reduces GSH concentration.^53^ On the other hand, lower MDA levels have been previously documented in the hippocampus during IRI in ischaemic rat brain following dexmedetomidine administration.^54^ Besides, a prior study also illustrated dexmedetomidine could diminish oxidative stress and downregulate caspase‐3 expression induced by IRI in skeletal muscle cells.^55^ Furthermore, suppression of ROS was associated with dexmedetomidine administration, which ultimately protects cardiomyocytes from mitochondria damage and cardiomyocytes apoptosis.^56^ The above‐mentioned evidence lends support to our findings, whereby dexmedetomidine decreased the levels of MDA, cleaved caspase‐3 and ROS and increased GSH levels.

Furthermore, additional experimentation in our study revealed that treatment with dexmedetomidine led to an increase in EGCs viability. Moreover, we came across a decrease in apoptosis as a result of dexmedetomidine treatment, as evidenced by reduced levels of the pro‐apoptotic proteins p65, p53, MnSOD, S100β, AIF, cleaved caspase‐3, Bax and increased levels of Bcl‐2 and GFAP. TERT expression reflects the level of the TERT protein in mitochondria, and the MnSOD, p65 and p53 levels were measured to evaluate the cellular response to oxidative stress.^57^, ^58^ In addition, AIF, cleaved caspase‐3, Bax and Bcl‐2 are widely used to evaluate the levels of cell apoptosis.^59^ According to Engelhard et al., apoptosis occurring in a rat cerebral I/R model after reperfusion can be prevented by administering dexmedetomidine, resulting in increased levels of Bcl‐2.^60^ The antiapoptotic proteins in the Bcl family, which includes both Bcl‐2 and Bcl‐XL, are also known to play important roles in the mitochondrial membrane to inhibit cell apoptosis by preventing the release of mitochondrial constituents into the cytoplasm. On the other hand, existing evidence suggests that the Bax protein is translocated from the cytosol to the mitochondrial membrane to induce cell death after the initiation of apoptotic signalling and undergoing a conformational change.^61^ Hence, the balance between Bax and Bcl‐2 represents one of the most critical factors in determining whether a cell will undergo apoptosis.^62^ It is also important to note that, dexmedetomidine is capable of preventing cortical neurons from undergoing apoptosis in vitro and in vivo.^63^ Furthermore, the data reported by Luo et al. indicates that dexmedetomidine reduces the percentage of apoptotic cells induced by hypoxia and ischaemia‐reperfusion.^64^

**FIGURE 6:**
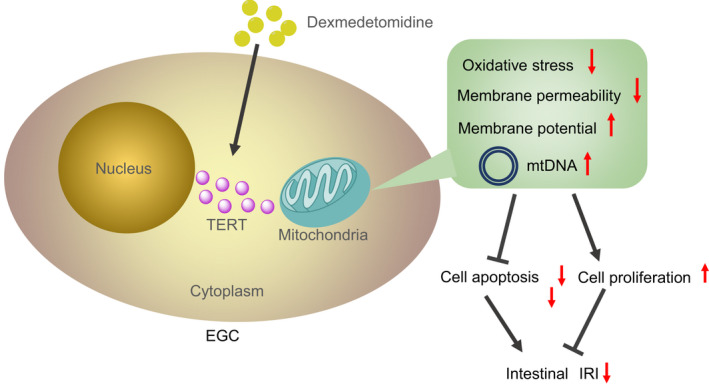
Schematic diagram of the mechanism by which dexmedetomidine affects intestinal IRI. Dexmedetomidine elevates the expression of TERT protein and facilitates its mitochondrial localization, reducing ROS and mitochondrial damage, thereby inhibiting EGC apoptosis caused by intestinal IRI

In conclusion, the key findings of our investigation provide evidence illustrating that TERT is downregulated in rat models with intestinal IRI. On the other hand, administration of various concentrations of dexmedetomidine exerted an augmenting effect on TERT expression to induce mitochondrial localization of TERT and attenuate oxidative stress (Figure 6). Our discoveries provide novel insights into the potential therapeutic use of treatments targeting mitochondrial localization, and oxidative stress in addition to how these prognostic markers could be applied in the setting of chemotherapeutic intestinal IRI treatment. However, the direct correlation between dexmedetomidine and TERT expression remains elusive and requires further elaboration in future studies. Additionally, it would also be prudent to employ specimens from IRI‐diagnosed patients for a comprehensive understanding of the specific mechanisms by which dexmedetomidine influences TERT in intestinal IRI.

## CONFLICT OF INTEREST

The authors have no conflicts of interest to declare.

## AUTHOR CONTRIBUTIONS

**Qian Hu:** Investigation (equal); Methodology (lead); Supervision (equal); Visualization (lead); Writing – review & editing (lead). **Xiao‐Ming Liu:** Conceptualization (equal); Formal analysis (equal); Resources (equal); Writing – original draft (equal); Writing – review & editing (equal). **Zheng‐Ren Liu:** Conceptualization (equal); Data curation (equal); Formal analysis (equal); Visualization (supporting); Writing – original draft (equal). **Zhi‐Yi Liu:** Data curation (equal); Investigation (equal); Validation (lead); Writing – review & editing (supporting). **Huai‐Gen Zhang:** Formal analysis (equal); Methodology (supporting); Software (equal); Writing – review & editing (supporting). **Qin Zhang:** Data curation (equal); Investigation (equal); Methodology (supporting); Writing – review & editing (supporting). **Yuan‐Lu Huang:** Data curation (equal); Software (equal); Supervision (equal); Writing – original draft (supporting). **Qiuhong Chen:** Data curation (equal); Resources (equal); Supervision (equal); Writing – original draft (supporting). **Wen‐Xiang Wang:** Data curation (equal); Resources (equal); Supervision (equal); Writing – original draft (supporting). **Xuekang Zhang:** Conceptualization (equal); Resources (equal); Writing – original draft (equal); Writing – review & editing (equal).

## Supporting information

Fig S1

Fig S2

Fig S3

Table S1

Table S2

Supplementary Material

## Data Availability

The datasets used and/or analysed during the current study are available from the corresponding author on reasonable request.
