# Supplementary figures and images for: Dexmedetomidine reduces enteric glial cell injury induced by intestinal ischaemia‐reperfusion injury through mitochondrial localization of TERT
Source: J Cell Mol Med. 2022 Apr 2;26(9):2594–606. doi: 10.1111/jcmm.17261 (PMC9077307; doi:10.1111/jcmm.17261)

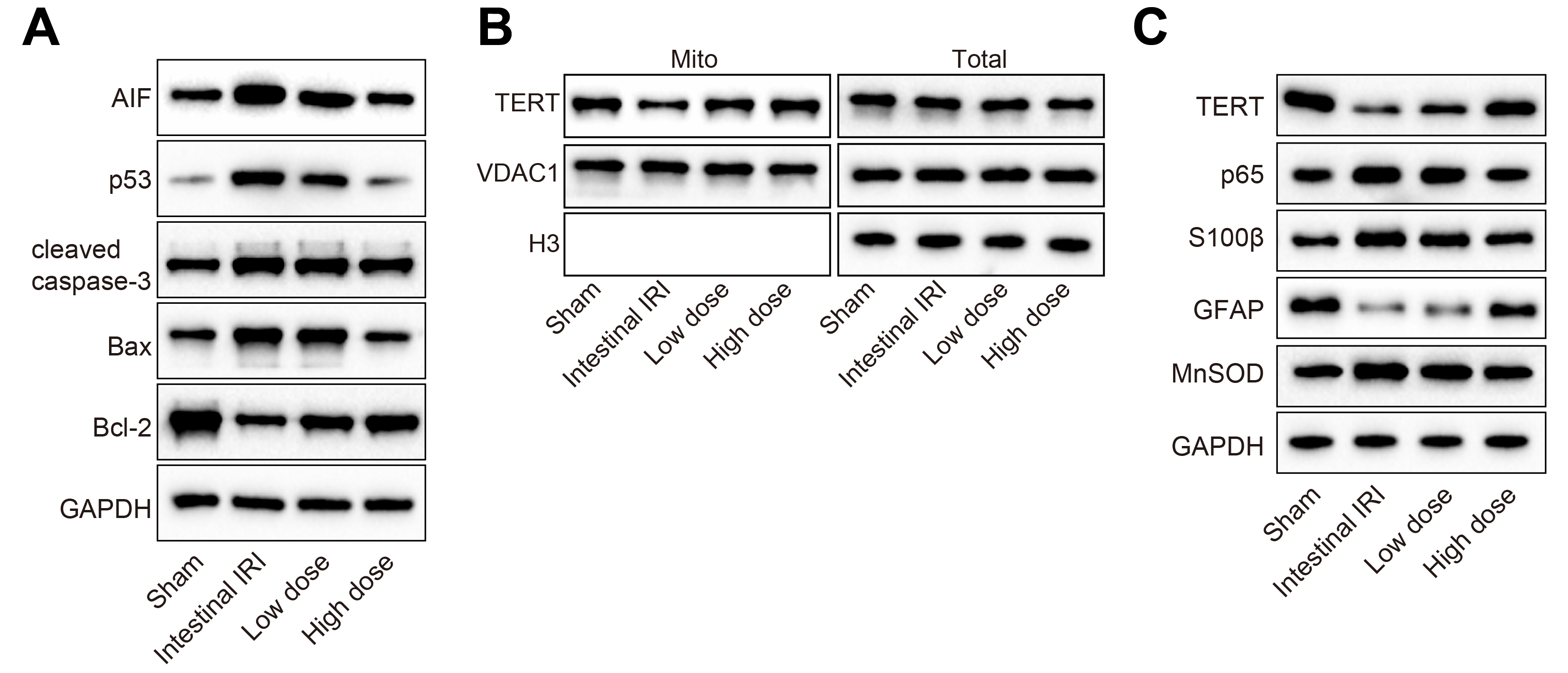

Supplement: Supplementary file 1 — Fig S1 [file JCMM-26-2594-s002.jpg]

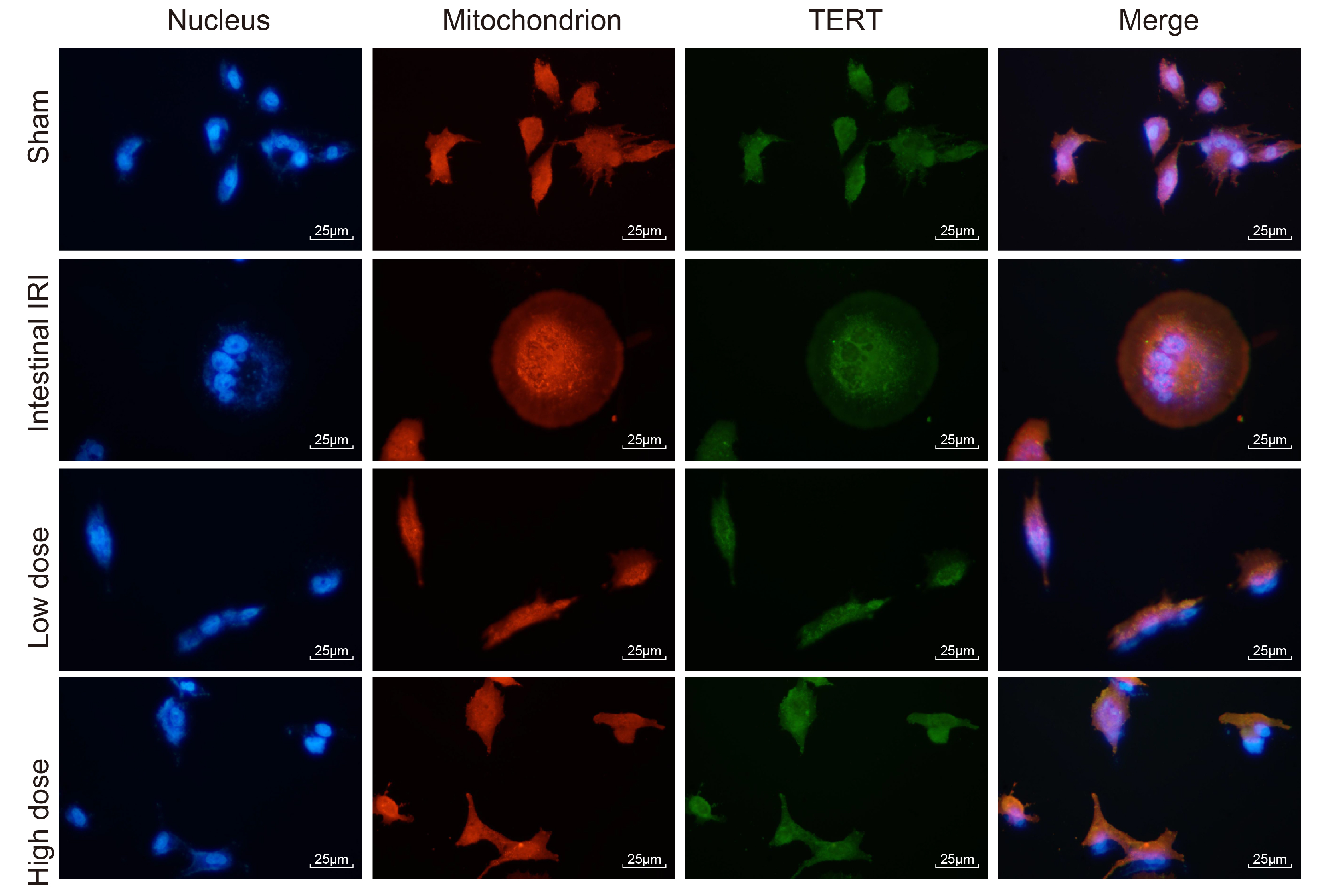

Supplement: Supplementary file 2 — Fig S2 [file JCMM-26-2594-s004.jpg]

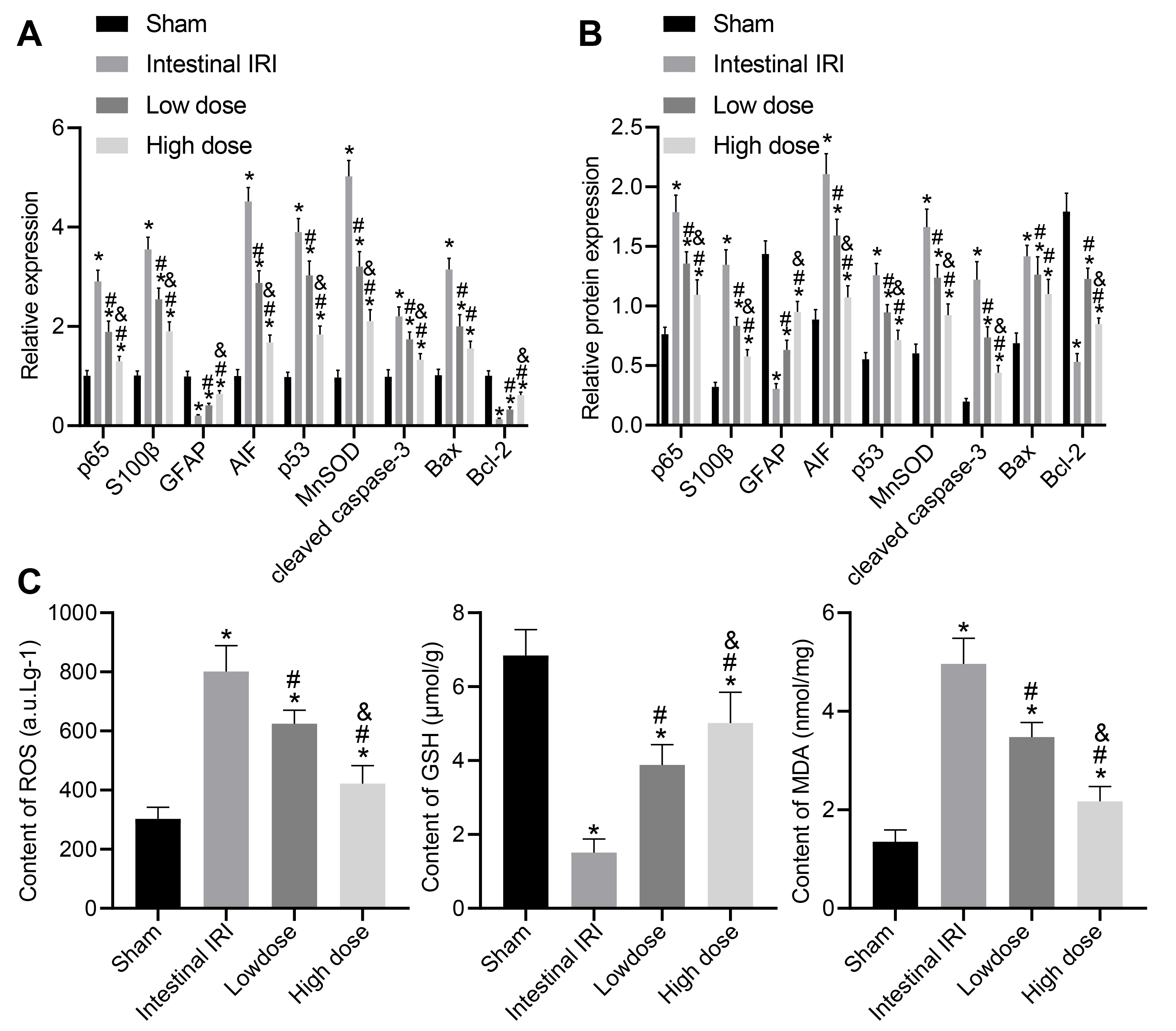

Supplement: Supplementary file 3 — Fig S3 [file JCMM-26-2594-s005.jpg]
