# Supplementary material for: Dexmedetomidine reduces enteric glial cell injury induced by intestinal ischaemia‐reperfusion injury through mitochondrial localization of TERT
Source: J Cell Mol Med. 2022 Apr 2;26(9):2594–606. doi: 10.1111/jcmm.17261 (PMC9077307; doi:10.1111/jcmm.17261)
Supplement: Supplementary file 4 — Table S1 [file JCMM-26-2594-s006.docx]

**TABLE** **S1** The primer sequences for RT-qPCR

| Gene | Sequence (5’ - 3’) |
| --- | --- |
| p65 | Forward: ATCTGTTTCCCCTCATCTTT  Reverse: CTGCGTCTTAGTGGTATCTGT |
| TERT | Forward: CTGTGCCTACCAGGGGAGAT  Reverse: CTGGCCCCAGTCAAGTTAGG |
| p53 | Forward: CATGTGCAACAGCTCCTGCATGG  Reverse: GTCAGCCCCACTTTCTTGATCAT |
| MnSOD | Forward: GGGCAAGGAGATGTTACAA  Reverse: GCTTGATAGCCTCCAGCAAC |
| S100β | Forward: AGAGGGTGACAAGCACAAGC  Reverse: CTTCCTGCTCCTTGATTTCCTCC |
| GFAP | Forward: GCGAAGAAAACCGCATCACC  Reverse: AAGGGAGAGCTGGCAGG |
| AIF | Forward: AGAAACTGGTGCCCTTGGTG  Reverse: GAGATGACACTGCACAACTGG |
| Bax | Forward: ATCCAGGATCGAGCAGGGAGGATGG  Reverse: AGATGGTCACTGTCTGCCATGTGGG |
| Bcl-2 | Forward: ATCAATGGCAACCCATCCTGGCACC  Reverse: AAACGCTCCTGGCCTTTCCGGC |
| GAPDH | Forward: CAGGAGAGTGTTTCCTCGTCC  Reverse: CAGGAGAGTGTTTCCTCGTCC |

Note: RT-qPCR, reverse transcription quantitative polymerase chain reaction; TERT, telomerase reverse transcriptase; MnSOD, manganese superoxide dismutase, superoxide dismutase 2; GFAP, glial fibrillary acidic protein; AIF, apoptosis inducing factor; Bax, Bcl-2 associated X; Bcl-2, B-cell lymphoma-2; GAPDH, glyceraldehyde-3-phosphate dehydrogenase
