# Supplementary material for: Dexmedetomidine reduces enteric glial cell injury induced by intestinal ischaemia‐reperfusion injury through mitochondrial localization of TERT
Source: J Cell Mol Med. 2022 Apr 2;26(9):2594–606. doi: 10.1111/jcmm.17261 (PMC9077307; doi:10.1111/jcmm.17261)
Supplement: Supplementary file 5 — Table S2 [file JCMM-26-2594-s001.docx]

**TABLE S2** Sequences for detection of mitochondrial common mutation levels

| Name | Sequence (5’ - 3’) | Final concentration (nmoL/L) |
| --- | --- | --- |
| mtDNA_4977bp | Forward: CCTTACACTATTCCTCATCACC | 100 |
|  | Reverse: TGTGGTCTTTGGAGTAGAAACC | 900 |
| COXI | Forward: TTCGCCGACCGTTGACTATTCTCT | 400 |
|  | Reverse: AAGATTATTACAAATGCATGGGC | 400 |
| β-actin | Forward: ACCCACACTGTGCCCATCTAC | 400 |
|  | Reverse: TCGGTGAGGATCTTCATGAGGTA | 400 |

Note: TEL, translocation Ets leukemia; COXI, cytochrome C oxidase subunit I.
