# Supplementary material for: Dexmedetomidine reduces enteric glial cell injury induced by intestinal ischaemia‐reperfusion injury through mitochondrial localization of TERT
Source: J Cell Mol Med. 2022 Apr 2;26(9):2594–606. doi: 10.1111/jcmm.17261 (PMC9077307; doi:10.1111/jcmm.17261)
Supplement: Supplementary file 6 — Supplementary Material [file JCMM-26-2594-s003.docx]

**SUPPORTING INFORMATION**

**FIGURE S1.** A, Representative image of Figure 2F. B, Representative image of Figure 3C. C, Representative image of Figure 5B.

**FIGURE S2.** Representative image of Figure 3B.

**FIGURE S3.** Dexmedetomidine reduces the expression of oxidative stress-related genes in the intestinal tissues of rats with IRI. A, The mRNA expressions of TERT, p65, MnSOD, S100β and GFAP in EGCs were determined by RT-qPCR. B, The protein expressions of TERT, p65, MnSOD, S100β and GFAP in EGCs were assessed by Western blot analyses. C, Determination of the levels of ROS, GSH and MDA in EGCs. *, *p* < 0.05 compared with the normal group; #, *p* < 0.05 compared with the intestinal IRI group; &, *p* < 0.05 compared with the low-dose group. The measurement data were presented as the mean ± standard deviation and compared using one-way ANOVA. The experiment was repeated 3 times.

**TABLE** **S1** The primer sequences for RT-qPCR

Note: RT-qPCR: reverse transcription-quantitative polymerase chain reaction; TERT, telomerase reverse transcriptase; MnSOD, manganese superoxide dismutase, superoxide dismutase 2; GFAP, glial fibrillary acidic protein; AIF, apoptosis-inducing factor; Bax, Bcl-2 associated X; Bcl-2, B-cell lymphoma-2; GAPDH, glyceraldehyde-3-phosphate dehydrogenase

**TABLE S2** Sequences for detection of mitochondrial common mutation levels

Note: TEL, translocation Ets leukaemia; COXI, cytochrome C oxidase subunit I.
